# Perceptions of Causes, Consequences, and Solutions of Intimate Partner Violence (IPV) in Mexican Women Survivors of IPV: A Qualitative Study

**DOI:** 10.3390/bs15060723

**Published:** 2025-05-24

**Authors:** Aniel Jessica Leticia Brambila-Tapia, Ignacio Brambila-Tostado, Martha Patricia Ortega-Medellín, Giovanna Georgina Ramírez-Cerón

**Affiliations:** 1 Departamento de Psicología Básica, Centro Universitario de Ciencias de la Salud (CUCS), Universidad de Guadalajara, Sierra Mojada #950, Colonia Independencia, Guadalajara 44340, Jalisco, Mexico; 2 Doctorado en Derechos Humanos, Centro Universitario de Tonalá (CUTONALÁ), Universidad de Guadalajara, Guadalajara 44340, Jalisco, Mexico; ignacio.brambila8345@alumnos.udg.mx; 3 Centro de Estudios sobre Aprendizaje y Desarrollo (CEAD), Departamento de Psicología Básica, Centro Universitario de Ciencias de la Salud (CUCS), Universidad de Guadalajara, Guadalajara 44340, Jalisco, Mexico; patricia.omedellin@academicos.udg.mx; 4 Instituto de Psicología y Educación Especial, Departamento de Psicología Aplicada, Centro Universitario de Ciencias de la Salud (CUCS), Universidad de Guadalajara, Sierra Mojada #950, Colonia Independencia, Guadalajara 44340, Jalisco, Mexico

**Keywords:** intimate partner violence, perceptions of causes, consequences and solutions

## Abstract

Intimate partner violence (IPV) is the main cause of violence against women, especially in Mexico. However, the causes, consequences, and solutions related to IPV have not been well understood in this population. A total of five focus groups that included Mexican women who were victims of IPV were conducted to understand the causes, consequences, and solutions related to IPV, and a thematic analysis was performed. A total of 32 participants were included, with a median (range) age of 35 (24–70). The main causes mentioned by the participants were (a) cultural ones, among which *Machismo*, transgenerational violence, and cultural norms and gender roles were the main categories; (b) emotional causes, among which the lack of emotional abilities and emotional dependence were the main categories; and (c) educative causes, among which the lack of information about mental health, emotional abilities, IPV, and healthy relationships was reported. The main consequences mentioned were (a) psychological; (b) physical; (c) economic; (d) family-related, including impacts on children; and (e) legal, in relation to IPV complaints and children’s custody. Finally, the main solutions mentioned by the participants were (a) prevention through education, including educational programs to address mental health, IPV information, healthy relationships, and training in emotional skills; (b) cultural transformation; and (c) institutional strengthening, with this last solution including improving public policies, improving legal advice, and training for legal authorities. In conclusion, the causes, consequences, and solutions related to IPV were varied and included many instances; therefore, its prevention and solution should be performed at the inter-institutional and community levels, in which the promotion of emotional skills should play a fundamental role.

## 1. Introduction

Intimate partner violence (IPV) is the most frequent type of violence against women worldwide (WHO, 2013), and it is a complex cross-cultural phenomenon defined as any behavior within an intimate relationship that causes harm (physical, psychological, sexual, or economic) to people within the relationship (García-Moreno et al., 2015).

In relation to the prevalence of IPV, according to Mexican government data from the National Institute of Statistics, Geography, and Informatics (INEGI, 2021), around 70% of women aged 15 years or older have experienced at least one violent incident during their lives, in any area, with psychological violence being the most frequently reported (51.6%), followed by sexual violence (49.7%), physical violence (34.7%), and, finally, economic/patrimonial violence or discrimination (27.4%). Specifically, IPV has been reported in 40% of Mexican women aged 15 years or older who are or have been in a relationship (INEGI, 2021). These data reflect the magnitude of the problem of gender violence and, specifically, IPV in Mexico. According to the United Nations Offices of Drugs and Crime (UNODC, 2019), IPV has been associated with femicides worldwide, with 47% of them being perpetrated by an intimate partner or a family member (UNODC, 2019).

The main risk factors associated with IPV in low-income and Hispanic countries are classified into socio-demographic, family-related, behavioral, and community factors (Gunarathne et al., 2023; Cummings et al., 2013). Among these, the following risk factors have been positively associated with IPV: a low socioeconomic level, attitudes that favor IPV, having been exposed to violence in childhood, having witnessed parental violence in childhood, alcohol and drug consumption, economic dependence, and low self-esteem. The main consequences are the impact on mental health and physical health and the impact on children (Gunarathne et al., 2023). Additional differences have been observed in IPV prevalence in the living areas of low-income countries; in a recent report, it was shown that urban slum areas have higher rates of IPV than non-slum urban areas or urban areas, which has been mainly explained by the controlling behavior by husbands (Chen et al., 2025).

With reference to international qualitative studies addressing IPV experiences and needs in affected women, we found some recent studies (Admassu et al., 2024; Gilliam et al., 2024; Wood et al., 2024; Sanchez et al., 2025) that explored the perceptions of women and other social actors with reference to IPV in different cultures and contexts, one of them including pregnant Mexican women (Gilliam et al., 2024). In these studies, the need for familiar and social support that IPV victims experience in order to seek help and escape the violence circle, as well as the negative impact that cultural norms exert in this kind of violence, are remarkable; similarities were observed in different countries such as Ethiopia and Mexico (Admassu et al., 2024; Gilliam et al., 2024). The experiences of Mexican women victims of IPV are strongly influenced by these norms, where the same family members “force” the victim to stay in a violent relationship by prioritizing the relationship with the woman’s partner, marriage, and family above women’s dignity (Gilliam et al., 2024).

The lack of social support is also evidenced in a qualitative study on abused women in rural areas of Canada (Wood et al., 2024), where the physical and cultural elements of rurality increase social isolation and coercive control and decrease women’s capacity for control over their life choices. In that study, norms and social expectations were also associated with the coercive control that they experienced. This coercive control is also related to intimate partner sexual violence; in this regard, a qualitative study explored the experiences of French women victims of this kind of violence (Sanchez et al., 2025) who reported complex emotional trauma as a consequence of this violence, which was difficult to identify and was also related to a traumatic childhood history and the idealization of romantic love. These studies report similarities among risk factors and personal experiences in relation to IPV in different countries, contexts, and cultures.

Qualitative studies performed in Mexico also indicate that *Machismo* is highly prevalent in this culture and is directly related to IPV (Gilliam et al., 2024; Agoff & Herrera, 2019). This important factor, along with the admixture of social isolation, traditional gender norms, and a lack of family ties and family support, contributes to this serious health and social problem in Mexican women (Agoff et al., 2007; Gilliam et al., 2024; Agoff & Herrera, 2019)

Concerning quantitative studies performed in Mexico, it was reported that some studies have addressed the factors associated with IPV and their consequences (Valdez-Santiago et al., 2006; Valdez-Santiago et al., 2013; Ávila-Burgos et al., 2009). These found that women being younger with less education, having two or more children in the household, consuming alcohol, and having a personal history of abuse in childhood were significant predictors of IPV. In addition, the partner’s alcohol consumption was the most important predictor of severe IPV (Ávila-Burgos et al., 2009). It was also found that in indigenous Mexican populations, the significant risk factors for IPV were a history of childhood abuse, working outside the home, the unemployment of the partner, and a high frequency of alcohol consumption by the partner (Valdez-Santiago et al., 2013). Another study performed among Mexican women users of the health system found that the emotional discomfort was significantly related to severe IPV (Valdez-Santiago et al., 2006).

Additionally, in a systematic review related to barriers to formal support for women who have experienced IPV in Spanish-speaking countries, which included nine studies performed in Mexico, the main barriers identified were intrapersonal, interpersonal, and organization-specific barriers. Among the intrapersonal barriers were shame and fear, a lack of knowledge about community resources and laws regarding IPV, and attitudes that favor IPV. The interpersonal barriers included partners’ threats, staying in the relationship for the children, the victims not wanting their family to know, and a lack of family support. The organization-specific barriers included themes such as mistrust in the police and the legal and health systems, along with poor experiences with reporting IPV in the past (Carney, 2024). Most of these factors are also related to reports on qualitative and quantitative studies in the Mexican population.

However, besides these reports on Mexican women victims of IPV, no studies were found in relation to the causes, consequences, and possible solutions of IPV in Mexican survivors. A study of IPV survivors’ perceptions of these themes is essential for a better understanding of the phenomenon of IPV. This understanding could lead to preventive and interventive programs for IPV and lead to changes in public policies that effectively prevent and treat the problem through linking the academic, social, and political sectors. Therefore, the objective of this study was to understand these perceptions in a sample of Mexican women survivors of IPV.

## 2. Participants and Methods

### 2.1. Ethical Considerations

This study was approved by the ethical committee of the Health Sciences University Center of the University of Guadalajara with approval number CI04724. All procedures were performed according to the Declaration of Helsinki guidelines.

All of the participants signed an informed consent form. This consent form included the invitation to participate in the study, the objectives, and the risks and benefits of participating. They were also told that they could abandon the study at any moment or not answer any question if they wanted. This consent form was signed by the participants with their first name only, which was used during the interviews. However, for data codification, all participants were identified with alphanumeric codes.

### 2.2. Participants

The inclusion criteria were the following: women older than 18 years old who were victims of IPV, visited the government offices in charge of dealing with this type of violence in the state of Jalisco (Secretary of Substantive Equality), lived in the city of Guadalajara or its surroundings, and agreed to participate in the study. The exclusion criteria were that the participants were not interested in participating, did not return a signed consent form, or did not finish the interviews.

### 2.3. Study Design

This is a descriptive qualitative study performed with an interpretive phenomenological approach. This approach was used because the objective of the study was to determine the perceptions and experiences of Mexican women survivors of IPV in their specific contexts (Palmer et al., 2010). The study was conducted within focus groups comprising 5 to 8 women, in which a list of guidance questions was made and discussed with the participants.

### 2.4. Procedures

Women who met the inclusion criteria were contacted by personnel of the Secretary of Substantive Equality and invited to participate in the study; women who agreed to participate were then contacted by the research team and interviewed in the facilities of the University of Guadalajara in groups of 5 to 8 women. To incentivize their participation, the possible participants were offered USD 25 to compensate for their transportation and food expenses.

There, they filled out an electronic questionnaire with their sociodemographic information; afterwards, they were interviewed using the focus group technique. The interview addressed the following themes: (a) the causes of IPV in Mexican women, which were investigated with questions such as “Which do you consider are the causes for a woman to be a victim of IPV in Mexico?”; (b) the consequences of IPV in Mexico, with questions such as “What have you experienced as a consequence of IPV?” and “What do you think will happen in the future with these consequences?”; and (c) the solutions to IPV, with questions such as “How could you have been prevented from being victims of IPV?”, “How do you think that IPV could be prevented at an institutional and personal level?”, “How do you think that consequences of IPV could be diminished?”, and “What do you think the government should do to treat these consequences?”.

### 2.5. Sociodemographic Variables

The following sociodemographic variables were measured: age, civil status, whether they had children, the number of children, schooling, whether they had a romantic partner, whether they were economically independent, the age of their union with the aggressor, their time of separation from the aggressor, and whether they had a job.

### 2.6. Data Analyses

Quantitative analyses: The quantitative description of sociodemographic variables included in order to characterize the sample was described with means and standard deviations, as well as medians and ranges for parametric and non-parametric distributions, respectively.

Qualitative analyses: Qualitative data were analyzed according to the thematic framework approach, for which all interviews were audio-recorded and transcribed verbatim; these transcripts were then coded into themes, subthemes, and codes according to the themes explored. At this point, the themes corresponded to the main themes about which the interviews were conducted, i.e., the causes, consequences, and solutions of IPV. The subthemes corresponded to the second-order categories and included broad categories that were independent from other similar categories and, at the same time, included sub-categories within them. These sub-categories were referred to as codes and were part of the third-order categories, which showed no further subdivisions. These analyses were performed by three members of the research team, who revised all of the transcriptions and proposed a classification of the obtained information into themes and subthemes, independently in the first step and together in the second step. To facilitate the analysis, a large matrix with all answers obtained from the participants was created by the three research team members. The data analysis aimed to identify all of the different answers given for each question (Gale et al., 2013). The code of each participant is given according to the number of their inclusion in the study; when we mention the quotes of the participants in the results, we also mention the age in years for that specific participant, e.g., the code “P1, 25y” refers to the first participant included, who was 25 years of age.

## 3. Results

A total of 95 women were invited to participate, of which 32 accepted; the median (range) age was 35 (24–70) years old, most of them (50.0%) were married, and half of these were separated. Most did not have a romantic partner (65.6%), did not have a job (59.4%), did not have economic independence (53.1%), and their schooling level was mainly secondary (34.4%). In addition, we observed that most of them joined their aggressor at a very young age of 20 years (13–29) and shortly thereafter began to experience IPV. We also observed that some of them still lived with their aggressors. These data are presented in Table 1.

The 32 women were interviewed in five focus groups.

### 3.1. Results of the Qualitative Analysis

A general overview of the qualitative analysis is presented in Table 2, where one can observe the themes, subthemes, and codes generated. We also added the number of participants of the 32 who were included that mentioned that specific answer.

### 3.2. Perceptions of Causes of IPV

Regarding the perceptions of the causes of IPV, six subthemes emerged: (a) cultural causes, (b) emotional causes, (c) educational causes, (d) socioeconomic causes, (e) family-related causes, and (f) addictions and substance abuse.

*Cultural causes:* The subtheme of cultural causes was, in turn, divided into the following three codes:

(a) Transgenerational violence: This refers to witnessing violence between one’s parents during childhood and having experienced it. Quote (P30, 34y): “If the son witnessed his father hitting his mother, then the son will do the same […], the upbringing from great-grandparents to the present is reflected”. (P6, 53y): “The mother (her mother in law), the advice she gave to both daughters and daughters-in-law was that, if you are about to answer you husband, take a mouthful of water and don’t speak”.
(b) Cultural norms and traditional gender roles: This code refers to the fact that women are in charge of domestic work and attend to the family’s men: (P13, 70y): “Since the 9 years my mom told me you are in charge of your brother’s clothes […] and I was a very beaten girl, precisely for that reason, because (according to my mother) I disrespected my brothers”. (P26, 30y): “Because at home I was taught the exact opposite, you shut up, obey, respect, and do what your partner does”.
(c) *Machismo*: This is the idea that men should command the relationship and have the right to control and subjugate women. The following quotes exemplify this: (P32, 52y): “*Machismo* has always existed and will never disappear […], right now we are here, in a little while they kidnap us, they kill us”. (P24, 56y): “It is the *Machismo* isn’t it? The fact that because men are men, they have the privilege to command, order or even hit”. (P9, 50y): “Today, there are women who stay at home doing housework and the man feels superior because of that and then his dominance begins, but when the woman goes out (to work) then a rivalry is established […], who pays is who commands”.

*Emotional causes*: This subtheme was divided into the following three codes:

(a) Lack of emotional abilities: This refers to a lack of self-esteem, fear of leaving one’s partner, and a lack of assertiveness in setting limits from the beginning of the relationship. Quote (P20, 34y): “At 16 you don’t know what a limit is, you don’t know any other love than one who comes and talks you nicely […] it’s said to be easy to set limits, but it is not because you are in love with that person […] as long as you don’t have the information, as long as you don’t have the psychological support, you will never understand what a limit is”. Quote (P3,45y): “At first one thinks it is normal […] that was my first mistake, I shouldn’t have get quiet since the beginning”.
(b) Emotional dependence: This refers to the emotional inability to leave one’s partner because of customs, need for affection, or fear of being alone. Quote (P6, 53y): “It is what hooks us and it is in the name of love that one endures everything”; (P5, 55y): “Before, even though he fought with me and no matter what he did to me and my children […] I forgave him and still felt save with him, I felt that he loved me”.
(c) Trauma and emotional deficiencies in childhood: This code refers to untreated trauma in childhood in relation to current emotional deficiencies, witnessing violence at home that was not solved with therapy, and the lack of a masculine figure during infancy, which is compensated with an intimate partner. Quote (P11, 25y): “Many women seek to fill the absence of a male figure, even when it means enduring violence, it’s as if we can’t let go of something we feel is necessary, even if it hurts us”; (P22, 24y): “If we were not given that love, we were not given that psychological attention since we were child, then obviously we grow up with those needs”.

*Educational causes:* This subtheme was divided into the following two codes:

(a) Lack of information about IPV and healthy relationships: This relates to providing information from childhood about what IPV is and how a healthy relationship should be. Quote (P2, 64y): “At beginning you are deeply in love and you think these are normal things […], one believes that it is normal, that one should be quite and endure”. (P6, 53y): “I didn’t know it was violence, until I went to therapy”.
(b) Lack of information about mental health and emotional abilities: This is exemplified in the following quote (P14, 29y): “One of the factors today is the lack of access to information about psychology and mental health, knowing our limits and letting other people know about their limits about others and how to recognize the toxicity of harmful actions and relationships”. Quote (P30, 34y): “As long as you don’t have the information, as long as you don’t have psychological support, you will never understand what a limit is”.

*Socioeconomic causes:* This subtheme was divided into the following two codes:

(a) Economic dependence: This code represents poverty and economic dependence on the partner. Quote (P9, 50y): “(The cause of IPV) It would be the economic dependence and the education”; (P2, 64y): “I couldn’t, I was stunned, I was scared, I was afraid. I couldn’t leave, I had no money […] he bought me clothes and all that stuff, it was total control”.
(b) Inequality in access to job opportunities: This code refers to the disadvantage of mothers with respect to men in getting a job, as exemplified in the following quote (P29, 28y): “The woman falls because she has to take care of the children because she has no financial support […], and even though you try and try, it is impossible with the schedules they set, with the transportation, the nannies”.

*Family-related causes*: This subtheme has the following two codes:

(a) Children’s dependence: This code refers to the fact that many women victims of IPV have children who depend on them, and they do not have the means to raise them. Quote (P28, 54y): “I never got away from it because I have two children and I stopped to think: where was I going to take them to live.? […]. I had never had a stable or good job, so I depended on the house and the money he gave me, there, education and financial independence would be what would help you get out of that”. (P29, 28y): “Because we have to save, take care of the children, because you don’t have financial support”.
(b) Social and familial judgments: This code refers to the lack of social support that many women victims of IPV experience, as exemplified by the following quote (P26, 30y): “Many times it is your own family or friends who tell you: not to be exaggerated or how toxic you are”. (P8, 43y): “I went with my relatives and it was worse, now I suffered violence from the family”.

*Addictions and substance abuse:* This subtheme is divided into the following two codes:

(a) Alcohol abuse: This code refers to the relationship between alcohol addiction and abuse through IPV, and it is examplified by the following quotes: (P14, 29y): “My mom has anxiety because she suffered trauma from my dad who liked alcohol […] she didn’t heal the trauma she had with my grandfather who was also alcoholic”. (P21, 30y): “He is a psychopath … and has taken psychiatric drugs … and also consumed alcohol, so he got really bad”.
(b) Drug consumption: This code refers to the relationship of drug addiction and consumption with IPV, as exemplified in the following quote: (P10, 25y): “My ex-partner is a drug addict and that triggered a violence that transformed him”. (P22, 24y): “He takes drugs even though he says he doesn’t and he became very aggressive”.

### 3.3. Perceptions of Consequences of IPV

The consequences of IPV were divided into six subthemes: (a) psychological consequences, (b) physical consequences, (c) social consequences, (d) economic consequences, (e) family-related consequences, and (f) legal consequences.

*Psychological consequences:* This subtheme was divided into the following two codes:

(a) Emotional disorders: This subtheme included anxiety, depression, suicide attempts, self-blame, suicidal ideation, panic attacks, and mental disorders such as posttraumatic stress disorder and borderline personality disorder. Quote (P26, 30y): “I suffer from insomnia, anxiety, and depression, I have suicide thoughts constantly, it is a constant fight. I’m on medication and therapy, but it was something that frustrated all my life”.
(b) Emotional isolation: This refers to the fact that victims of IPV are isolated from their support network by fear of their partner, by being locked in the house doing housework, and by the inability to trust people. Quote (P27, 29y): “I became very afraid of going out on the street because of his threats, that made it impossible for me to have a stable job … I lost all my friends”. (P6, 53y): “I couldn’t go to visit anyone, I had to stay at home … the neighbors didn’t know me … all my emotions were silent”.

*Physical consequences:* This subtheme refers to the physical harm and diseases that the violence triggered in the victims; this subtheme was divided into the following two codes:

(a) Physical injuries: This is exemplified in the following quotes: (P17, 24y): “My last partner threw me while I was pregnant. I spent two months in the hospital”. (P6, 53y): “He hit me on my arms. They were so bruised, I couldn’t move them”.
(b) General health effects: This code refers to the appearance of psychosomatic illness or worsening of physical illness because of the stress experienced due to IPV. Quote (P2, 64y): “I feel like all of that was landing the diseases on my body”. (P6, 53y): “I also became physically ill”.

*Social consequences:* This subtheme was divided into the following two codes:

(a) Stigmatization: This refers to criticism and social judgment for being a victim of IPV. Quote (P28, 54y): “My son asked me how was it possible that I was not able to get him [my husband] out of the house [with a complaint] … because I am afraid and shame with others, that noticed it”; (P5, 55y): “I blinded myself and said to myself I am crazy […] well, through religious situations one makes the decision to forgive and to fall into the same thing again”.
(b) Breaking of social relationships: This refers to the loss of one’s social network because of violence. Quote (P29, 28y): “It broke my structure, I had to change my friendships, my job, and residency, emotionally and structurally it did break me all over”. (P11, 25y): “My work and social life declined, I had to move to a new workplace, and most of my friends stopped talking to me”.

*Economic consequences:* This subtheme has the following three codes:

(a) Loss of resources: This code refers to the loss of personal belongings, documents, and the house where the victim lived together with the partner. Quote (P28, 54y): “I experienced all kinds of violence, the last thing he did was to break furniture, the dresser, the closet, he destroyed everything”. (P32, 52y): “He had the nerve to throw away my clothes, he ransacks my closet, take my documents and now he is the one asking me for a compensation”.
(b) Loss of job opportunities: This code refers to the loss of job opportunities as a consequence of IPV; this is because the victim had to change her job, because she had mental health conditions that prevented her from having a job, or the aggressor did not allow the victim to work. Quote (P26, 30y): “To find a job with depression and anxiety I have to knock on many doors”. Quote (P6, 53y): “(He) did not want me to work”.
(c) Being economically independent: This code refers to the need to be economically independent after an IPV experience. The following quotes exemplify this: (P2, 64y): “To get a job as an elderly woman, it is not so easy”; (P3, 45y): “My satisfaction is having started working at 45 years old without work experience”.

*Family-related consequences:* This subtheme was divided into the following two codes:

(a) Impact of IPV on the children: This code refers to the physical and psychological impact of IPV on children. The following quotes exemplify this: (P13, 70y): “My daughter (of 45 years old) told me: it is that my dad is harassing me”; (P23, 35y): “My son started acting violently towards his siblings, toward me and his father”; (P30, 34y): “My daughter was badly beaten because her father hit her”, “My daughter doesn’t show feelings, she doesn’t show love, because of everything she lived with her father”.
(b) Loss of contact with their children: This code refers to the fact that many aggressors take children away from their mothers using lies and economic influence, as exemplified in the following quote: (P30, 34y): “They are going to adjust two years that I have not seen my son because his father took him away from me alleging that I have childish mistreatment towards him”; (P21, 30y): “He takes my daughter away and I am devastated. I didn’t see my daughter for 4 months”.

*Legal consequences:* This subtheme was divided into the following two codes:

(a) Ineffectiveness of the legal system: This code refers to delayed legal processes and failures in laws: (P27, 29y): “When I started to investigate why the processes were not moving forward I realized that many processes do not move forward because the laws that should protect us are poorly written, so that makes it impossible for the prosecutor’s office and the people who are there to assist us, to do justice”. Quote (P17, 24y): “They were going to give her protective measures but they never went to check her home … They found her dead”.
(b) Legal impunity: This code refers to the legal impunity that perpetrators of IPV have in the Mexican justice system, while victims lack protection and an opportunity to repair the damage: (P27, 29y): “They feel untouchable, they say: I did her of everything and all ended up with 3000 pesos … so there really is no tangible consequence”. (P26, 30y): “A year later (to file a complaint) they may be call you and tell you *The file wasn’t processed* […] the aggressor is still out there, he is a predator […] they are not protecting us from the aggressor.”

### 3.4. Perceptions of Solutions to IPV

The perceptions of solutions to IPV were divided into five subthemes: (a) prevention through education, (b) psychological support, (c) institutional strengthening, (d) women’s empowerment, and (d) cultural transformation.

*Prevention through education:* This subtheme was divided into the following four codes:

(a) Educational programs in schools: This code refers to the need for education about violence and IPV, as well as the development of skills in schools: (P10, 25y): “If schools talked about this, children would identify what is wrong, not only with women but with how we are treated overall”. (P14, 29y): “That it was an extra class on managing emotions … setting limits … managing the economy”.
(b) Training in personal skills: This code refers to the need to increase emotional skills in children and adults: (P10, 25y): “To perform a course to train girls to feel able to be alone, without the ideology that a man is going to come and help her”; (P9, 50y): “I have to set limits and you have to set your limits too (in a relationship)”.
(c) Culture of peace at home: This code refers to the importance of education against violence at home: (P8, 43y): “The respect for all live beings […] and public spaces […] to create a better society”. (P29, 28y): “From homes, values could be taught”.
(d) Preventive programs in social media: This code refers to carrying out social media programs to prevent IPV. Quote (P13, 70y): “This should be preventative, I return to the mass media like television, to give information about IPV”. (P5, 55y): “Those programs focused on prevention be made … such as radio and television”.

*Psychological support:* This subtheme was divided into the following two codes:

(a) Psychological prevention: This code refers to the need for psychological studies of couples before they marry to identify violent behaviors and the need for psychological therapy from childhood. Quotes: (P23, 35y): “It would be great that by law, they were required to undergo a psychological study at the time of getting married”; (P14, 29y): “That the child from an early age has psychological therapy, and the parents have it at the same time”.
(b) Psychological therapy: This code refers to the need to treat personal and relationship issues with a psychologist. Quote (P23, 35y): “When we are in a relationship one must undergo personal and couple therapy”. (P11, 25y): “To start a new relationship, you must to heal the first one because that creates a lot of insecurity. You must to work on your self-esteem to see that you are worth a lot. We are not an object. We are a human being who is worth a lot”.

*Institutional strengthening:* This subtheme was divided into the following three codes:

(a) Improving legal advice: This refers to the need to improve legal information in governmental institutions related to divorce and IPV complaints. Quotes (P22, 24y): “I would like to know how far I can get out of there, how far and why I can run from there”; (P9, 50y): “The last advisor I had is not qualified, as if he doesn’t want to move forward in the process”.
(b) Training for legal authorities: This code refers to the importance of training police and legal staff in IPV situations, increasing empathy, and simplifying processes. Quote (P27, 29y): “They (the authorities) should be more empathic. When one goes to report, the same women treat the woman who is going to make the report badly. They also demand too much. If you go with a bruise on your face, a bruise on your arm, or a bruise on your leg, it is very little to file a complaint. You must to go almost with one foot in the box so that they can accept your complaint”. Quote (P9, 50y): “There should be more and better trained (victims assistance centers)”.
(c) Improving public policies: This code refers to improving public policies related to IPV. Quote (P29, 28y): “Since laws are not very specific and clear, the institutions cannot do anything … For example, the law against vicarious violence was just recently approved and there is not even a route, there is no access route”; (P26, 30y): “The law is more on the side of men”.

*Women’s empowerment:* This subtheme was divided into the following three codes:

(a) Economic independence: This code refers to the need to promote economic independence from a young age. Quote: (P10, 25y): “Some kind of help, that there is a course where we are thought how to generate, it would have been different …”; (P28, 54y): “It would be the education, the economic independence that would help you to get out of that”.
(b) Support networks among women: This code refers to the need to increase the number of women’s support groups. If there were more groups of women sharing experiences, many would feel encouraged to leave these situations. Quote (P2, 64y): “In the workshops we have done, we have formed real support groups”. (P11, 25y): “Part of raising awareness has to do with support networks, that we all have a support network where we feel safe”.
(c) Leaving an aggressive partner: This code refers to the fact of leaving one’s partner. Quotes (P16, 35y): “I would say it’s better to stay away from the aggressor, because he will always be there, abusing you”. (P30, 34y): “So after four (suicide) attempts, I said well … what do I have to do? To get away, its over”.

*Cultural transformation:* This subtheme was divided into the following three codes:

(a) Changing gender roles: This code refers to the change in the concept that men are the head of the family. Quote: (P8, 43y): “Many times the society makes them like that […] they say a man is strong and don’t cry, a man hits, many women are more sexist [than men], they (men) can also be creative, be kind, cry”. (P16, 35y): “That has my mother, that makes us women see as we are ones obliged to help at home and that men are not obliged to do it, so they have a *machismo*”.
(b) Men can also suffer from IPV: This code refers to the fact that, due to traditional gender roles, many men that suffer from IPV from a woman do not report it. Quote (P31, 49y): “I know a person that is violented by his wife”.
(c) IPV with sexual diversity: This code refers to the lack of help groups for women in homosexual relationships who have been abused and the lack of availability of groups for aggressive women in institutions. Quote: (P14, 29y): “My psychologist told me: You know that I have 4 more cases plus yours of (aggressive) girls (in homosexual relationships)”.

## 4. Discussion

In Mexico, IPV is a serious social problem, and it is an important cause of a social and mental health burden (Medina-Nuñez & Medina-Villegas, 2019). In this qualitative research, we addressed the causes, consequences, and solutions related to IPV in Mexican women survivors, and we found that many factors previously associated with IPV appeared in our interviews, including the following: exposure to violence in childhood, having witnessed parental violence in childhood, alcohol and drug consumption, sexist ideas, economic dependence, and low self-esteem (Gunarathne et al., 2023; Cummings et al., 2013). These causes also coincide with the risk factors reported in studies performed in Mexico, in which childhood abuse and alcohol consumption were associated with IPV (Ávila-Burgos et al., 2009; Valdez-Santiago et al., 2013; Carney, 2024). These factors have also been associated with IPV in pregnant Mexican women, where the presence of emotional distress, being single, being unmarried, and living with the partner were also reported (Doubova et al., 2007).

Likewise, the main previously reported consequences were also found in this study: the physical and mental impact and the impact of IPV on children; these have been reported at the international level (Gunarathne et al., 2023) and at the national level (Valdez-Santiago et al., 2006).

However, other non-reported causes and consequences were found. In this sense, although most of the results coincide with previously reported information (mainly with reference to the causes and consequences of IPV), the description of how these factors relate to the phenomenon was better explained within these interviews. In addition, new subthemes regarding causes and consequences emerged during the interviews, including “emotional causes” and “educative causes” and, regarding the consequences, including “social consequences”, “economic consequences”, “family-related consequences”, and “legal consequences”. Furthermore, the solutions that the IPV victims suggested have not been previously explored qualitatively or quantitatively.

Regarding the causes, it is interesting that *Machismo* was the main cause of IPV mentioned by the participants; this term refers to the stereotype of alpha males in Latin culture and encompasses the qualities of virility, bravado, and being the decision maker of the family (Cummings et al., 2013). This variable has been associated with IPV in Latin cultures, where the rates of IPV are disproportionately higher than in non-Latin cultures (Cummings et al., 2013; González-Guarda et al., 2010; Caetano et al., 2004; Aldarondo et al., 2002). *Machismo* has also been related to drinking alcohol and related risky behaviors (González-Guarda et al., 2009), which are also risk factors for IPV (Cummings et al., 2013; González-Guarda et al., 2010). Additionally, this cause (*Machismo*) has been reported as a highly prevalent risk factor for IPV in Mexican survivors (Gilliam et al., 2024; Agoff & Herrera, 2019); this risk factor is also related to cultural norms and gender roles in this and other studies performed in Mexico (Gilliam et al., 2024; Agoff & Herrera, 2019; Carney, 2024), where the victim’s family judges her when she wants to get out of a violent relationship (Gilliam et al., 2024) and persuades her not to seek help (Carney, 2024). All of these cultural factors are related to “transgenerational violence”, the third code of the “cultural causes” observed in this study; this is defined as the repetition of violent behavior across generations, so a violent behavior that a woman has experienced or learned as “normal” is repeated with her new family members. Likewise, the violent behavior that a man experiences or observes in his parents’ relationship is replicated with his wife and even children, a point that was specifically mentioned by participants.

The “cultural causes” also relate to specific consequences, which include “emotional isolation” and “stigmatization”, as well as the solution of social support (code: support networks among women). Social isolation and stigmatization have also been mentioned in reports on women victims of IPV in Mexico and other countries (Carney, 2024; Wood et al., 2024; Admassu et al., 2024; Agoff & Herrera, 2019; Agoff et al., 2007) and represent an important barrier to seeking help (Gilliam et al., 2024). Social isolation is also related to a higher risk of coercive control, which has been observed in rural areas of Canada (Wood et al., 2024), and it was also linked with the cause of “economic dependence” in this study.

In line with these “cultural causes”, femicides are reported at a higher frequency in Latin countries, with Mexico being in second place for femicides in Latin America and the Caribbean (OIG, 2023). It has been shown that Hispanic women are at twice the risk of being killed by a partner than non-Hispanic women in the US (Azziz-Baumgartner et al., 2011). These differences between Hispanic/Latin cultures and non-Latin cultures could be attributed to the cultural influence of *Machismo* and its detrimental consequences. In this sense, although *Machismo* has not been researched in relation to femicides, a report on men who committed femicide in South Africa showed that among the causes related to this behavior was the attempt to perform exaggerated versions of predominant ideals of masculinity, emphasizing extreme control and dominance over women (Mathews et al., 2014). This ideology is part of the definition of *Machismo*. The relationship between *Machismo* and femicides was also found in the following quote from a participant (P32, 52y): “*Machismo* has always existed and will never disappear […], right now we are here, in a little while they kidnap us, they kill us”.

The presence of *Machismo* as a cultural cause of IPV can be addressed with the proposed solution of “cultural transformation”, specifically by “changing gender roles”. This solution could be implemented at home, in schools, and on social media, and it is linked with the proposed solution of “prevention through education”. In this sense, to the best of our knowledge, no preventive programs have been implemented in schools in Mexican society; therefore, the implementation of preventive programs addressing themes such as equal gender roles at home from childhood, healthy relationships, early detection and treatment of IPV, and the eradication of *Machismo* could diminish the cultural causes of IPV. These preventive programs should be evaluated and constantly improved.

Another important and frequently mentioned cause of IPV that was not previously reported was “emotional causes”, where the lack of emotional abilities, including self-esteem and assertiveness, was related to being an IPV victim. This cause could be addressed with the proposed solution of “training in personal skills” within the subtheme of “prevention through education”. In this sense, few studies related to intervention programs for IPV were found (Alsina et al., 2023), and in those studies, none of the interventions explored included training in emotional abilities from childhood to prevent IPV; most of them were implemented for women who had suffered from IPV or were at high risk of suffering from it. In this sense, we found one study that associated emotional abilities with a positive affect and future orientation in a population of victims of IPV (Cabras et al., 2020). Additionally, self-esteem and social problem-solving styles were associated with IPV victimization in a French sample of emerging adults (Cherrier et al., 2023). This represents an opportunity to prevent IPV by fomenting psychological abilities in children, which, in addition to preventing IPV, could improve the mental health of the population and diminish other types of violence. The lack of emotional abilities could also be linked to other causes of IPV mentioned by the participants, including emotional and economic dependence.

**Figure 1:**
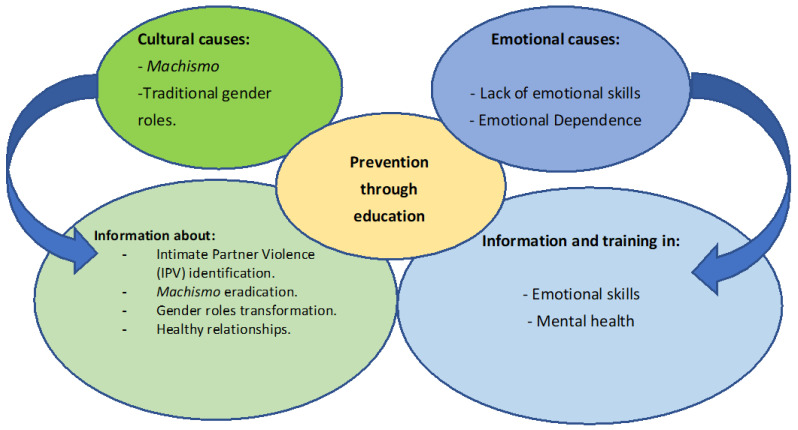
Main causes and solutions of IPV.

The two main causes and solutions mentioned by the participants are summarized in Figure 1.

Regarding the consequences of IPV, the results emphasize the psychological and physical consequences; in this sense, anxiety, depression, suicide attempts, and mental disorders, including post-traumatic stress disorder (PTSD), have been previously documented as consequences of IPV (Gunarathne et al., 2023). Therefore, one of the solutions to IPV mentioned by the participants was psychological therapy. In this regard, although government institutions offer 12 free sessions of psychological therapy, the victims stated that this is not enough and that more free sessions should be offered.

Psychological consequences in children were also mentioned; in children, violent behaviors and mental health problems have been reported, and these findings coincide with those of previous studies, in which psychological and behavioral effects, as well as developmental impairment, have been observed in children of women victims of IPV (Pahn & Yang, 2021; Abel et al., 2019). These important consequences in children contribute to perpetuating the cycle of violence and often remain unaddressed. In this sense, government institutions should also offer psychological treatment for children of IPV victims, which is currently not available in institutions that deal with IPV. In addition, a family-related consequence of IPV mentioned by the participants was the loss of contact with their children. In these cases, the participants mentioned that legal trials can last for years and, in the meantime, they do not see their children for a long time or only in supervised visits, which adds to the fact that legal advice and services are not provided by government institutions. Therefore, institutional strengthening by improving legal advice and public policies that truly provide justice and protect the victims was mentioned as a solution to IPV.

Additionally, the training of legal authorities to better treat the cases of IPV was also mentioned because many IPV victims are re-victimized during the process of filing a complaint of violence, making this a very slow and complicated process in which the victims are not believed. Therefore, the proposed training should include an increase in empathy from legal staff and simplification of the process, which should be performed with greater speed and efficacy. This ineffectiveness of the legal system could also explain the high rates of femicides reported in Mexico, a country with high levels of corruption and impunity (Universidad de las Américas, 2022). The impunity and failures in the legal system were also mentioned in previous reports in Ethiopia and Latin American countries (Admassu et al., 2024; Carney, 2024), where injustice or minimal punishment for aggressors (Admassu et al., 2024) and no legal deterrent for IPV perpetrators were observed (Carney, 2024). Finally, it is important to emphasize the codes within the subtheme of “cultural transformation”, which include the fact that men can also be victims of IPV by women and that women can also be aggressors of IPV in homosexual relationships; these variations in IPV should be also contemplated in preventive and treatment programs for IPV. In this regard, a recent report on a population of LGB victims of IPV in Turkey found that this population requires special needs that should be considered (Ummak et al., 2025).

This study had the following limitations: the rate of acceptance for participation was 1:3, which means that one-third of the invited women agreed to participate; this could represent a bias by considering that women who agreed could have different experiences from those who did not agree, and the results could encompass the main perceptions of this population. Another limitation was the fact that some participants were already using psychotherapy, while others were not or had never used it. All of these characteristics made the group more heterogeneous and could have affected the results.

In conclusion, the perception of Mexican women victims of IPV regarding the causes, consequences, and possible solutions of this violence was varied. The main causes mentioned were cultural, emotional, educational, socioeconomic, and family-related causes and substance abuse, while the main consequences were psychological, physical, economic, and family-related consequences, including effects on children and legal consequences. The solutions proposed mainly referred to preventive programs implemented through education, psychological support, institutional strengthening, women’s empowerment, and cultural transformation. To achieve these objectives, it is necessary to have cooperation among governmental institutions, academia, and society. This cooperation can be achieved with the development of academic studies such as the present one and their presentation to legal authorities in charge of IPV, which could improve the preventive and intervention programs for this population. Finally, the action of politicians who can make effective changes in public policies is also necessary.

In addition, the inclusion of civil organizations related to IPV is essential because they know the main needs and solutions for this important problem; therefore, the linkage between proposals made by researchers and civil society with government institutions can be the first step in making an effective change in IPV. However, political commitment is crucial; in this sense, a recent report showed that the political commitment in the Mexican health system to address violence against women reflects limited efforts and capacity to address this problem (Morse et al., 2025). This emphasizes the need to increase societal and academic cooperation in order to perform the required actions.

Although these results coincide with previous reports in different populations concerning the causes and consequences of IPV, new causes, consequences, and solutions that were not previously mentioned emerged in this study, especially the theme of training in emotional abilities, which could be addressed in further preventive and intervention programs to diminish IPV and its consequences and may be particularly relevant to the Mexican population.

## Figures and Tables

**Table 1:** Sociodemographic characteristics of the participants.

Variable	Descriptive Data (*n* = 32)
Age, median (range)	35 (24–70)
Civil status, *n* (%)	
- Single	10 (31.2)
- Married, separated	8 (25.0)
- Married, not separated	8 (25.0)
- Free union	3 (9.4)
- Divorced	3 (9.4)
- Widow	0 (0.0)
With romantic partner, *n* (%)	11 (34.4)
With children, *n* (%)	31 (96.9)
With job, *n* (%)	13 (40.6)
With economic independence, *n* (%)	15 (46.9)
Schooling, *n* (%)	
- Elementary school	3 (9.4)
- High school	11 (34.4)
- Preparatory	10 (31.3)
- Bachelor’s degree	7 (21.9)
- Master’s degree	1 (3.0)
- Doctor in Philosophy (Ph.D.)	0 (0.0)
Age of union with the aggressor (years), median (range)	20 (13–29)
Age at which they started to experience violence, median (range)	21.5 (13–30)
Time of separation from the aggressor (years), median (range)	0.5 (0–22)

**Table 2:** Themes and subthemes of the focus groups.

Perceptions of Causes of IPV	Perceptions of Consequences of IPV	Perceptions of Solutions to IPV
**Cultural causes:** (a) Transgenerational violence (6/32). (b) Cultural norms and gender roles (5/32). (c) *Machismo* (11/32).	**Psychological consequences:** (a) Emotional disorders (18/32). (b) Emotional isolation (6/32).	**Prevention through education:** (a) Educational programs in schools (8/32). (b) Training in personal skills (8/32). (c) Culture of peace at home (2/32). (d) Preventive programs in social media (2/32).
**Emotional causes:** (a) Lack of emotional skills (9/32). (b) Emotional dependence (4/32). (c) Trauma and emotional deficiencies in childhood (4/32).	**Physical consequences**: (a) Physical injuries. (9/32). (b) General health effects (3/32).	**Psychological support:** (a) Psychological prevention (2/32). (b) Psychological therapy (17/32).
**Educative causes:** (a) Lack of information about IPV and healthy relationships (4/32). (b) Lack of information about mental health and emotional abilities (2/32).	**Social consequences:** (a) Stigmatization (2/32). (b) Breaking of social relationships (3/32).	**Institutional strengthening:** (a) Improving legal advice (2/32). (b) Training for legal authorities (5/32). (c) Improving public policies (3/32).
**Socioeconomic causes:** (a) Economic dependence on the partner (2/32). (b) Unequal access to job opportunities (1/32).	**Economic consequences:** (a) Loss of resources (3/32). (b) Loss of job opportunities (7/32). (c) Being economically independent (5/32).	**Women’s empowerment:** (a) Economic independence (2/32). (b) Support networks among women (4/32). (c) Leaving the aggressive partner (3/32).
**Family-related causes:** (a) Children’s dependence (5/32). (b) Social and family judgments (4/32).	**Family-related consequences:** (a) Impact of IPV on the children (4/32). (b) Loss of contact with their children (4/32).	**Cultural transformation:** (a) Changing gender roles (3/32). (b) Men can also suffer from IPV (2/32). (c) IPV with sexual diversity (1/32).
**Addictions and substance abuse:** (a) Alcohol abuse (4/32). (b) Drug consumption (5/32).	**Legal consequences:** (a) Ineffectiveness of the legal system (12/32). (b) Legal impunity (4/32).	

IPV: intimate partner violence. The number of participants out of the 32 included who mentioned that specific answer is shown in parentheses.

## Data Availability

The raw data supporting the findings of the manuscript are available from the corresponding author upon reasonable request.
